# Endowing iPSC-Derived MSCs with Angiogenic and Keratinogenic Differentiation Potential: A Promising Cell Source for Skin Tissue Engineering

**DOI:** 10.1155/2018/8459503

**Published:** 2018-09-13

**Authors:** Weimin Lin, Miao Chen, Chen Hu, Siyu Qin, Chenyu Chu, Lin Xiang, Yi Man, Yili Qu

**Affiliations:** ^1^State Key Laboratory of Oral Diseases, West China Hospital of Stomatology, Sichuan University, Chengdu 610041, China; ^2^Department of Oral Implantology, West China Hospital of Stomatology, Sichuan University, Chengdu 610041, China

## Abstract

Induced pluripotent stem cells (iPSC) hold tremendous potential for personalized cell-based therapy for skin regeneration. Aiming to establish human iPSCs as a potential cell source for skin tissue engineering, we expect to obtain an epidermal-like cell line with angiogenic and keratinogenic differentiation potential via inducing iPSC-derived mesenchymal stem cells (iPSC-MSCs) with basic fibroblast growth factor (bFGF) and/or keratinocyte growth factor (KGF). The results show that iPSC-MSCs were successfully induced with a positive FGFR/KGFR expression on the cell surface. BFGF/KGF induction could significantly increase the expression of vascularization marker CD31 and keratinization marker CK10, respectively, while when combined together, although CD31 and CK10 were still positively expressed, their expressions were lower than that of the single induction group, suggesting that the effects of the two growth factors interfered with each other. This cell line with angiogenic and keratinogenic differentiation potential provides a promising new source of cells for the construction of well vascularized and keratinized tissue engineered skin, furthermore establishing an effective strategy for iPSC-based therapy in skin tissue engineering.

## 1. Introduction

Skin transplantation is one of the main methods to treat skin defects such as extensive burns and refractory skin ulcers. However, the source of skin grafts is limited and there is a greater damage to the donor area. In recent years, with the rise of tissue engineering, tissue engineered skin is considered to have the unique advantage of repairing damaged tissue and possibly exercising part of the skin function.

Mesenchymal stem cells (MSCs) are one of the most commonly used cell sources in tissue engineering due to their multipotential differentiation potential. Nevertheless, autologous MSCs are lack of in vitro expansion capacity, and their application is limited by patient age and some other health conditions [1, 2]; thus there is a need for a more stable and less restrictive cell source for tissue engineering.

IPSCs have been shown to differentiate into multiple cell types, providing attractive prospects for personalized therapy. However, one of the great limitations to the clinical application of iPSCs is their tumorigenicity. To limit the undesired tumorigenesis associated with iPSC pluripotency, in vitro differentiation of iPSCs into downstream cells before clinical application is considered to be necessary [3]. IPSC-derived cells have been demonstrated to be not immunogenic to the isogenic or autologous immune system [4, 5]. The iPSC-MSCs exhibited greater proliferation potential than other traditional sources of MSCs. Tissue-specific, senescence-associated, and age-related DNA methylation patterns were erased during reprogramming [6, 7]. Unlike bone marrow MSCs, iPSC-MSCs that could be subjected to long-term culture without resulting in explicative senescence [8]. In addition, iPSC-MSCs displayed immunomodulatory functions similar to those of MSCs and could suppress the rejection from immune system [9]. Therefore, differentiation of iPSCs into iPSC-MSCs before clinical applications might be a more feasible and efficient strategy.

Vascularization and epidermal keratinization are important for ideal healing of the wound. Firstly, angiogenesis is a key step in the wound healing process, providing oxygen and nutrients for the defect area, while taking away metabolites to promote immune response and tissue regeneration. Meanwhile, keratinization of renascent skin is also critical: the keratin layer can resist external frictional stimuli and bacterial intrusion, which plays an important role in the quality of wound healing. Therefore, the construction of a cell line with the potential of vascularization and keratinization has an important significance for skin tissue engineering.

Cytokines are involved in cellular communication for regulating the differentiation and plasticity of MSCs. Fibroblast growth factor (FGFs) family is a kind of multifunctional growth factor; its effective application can restore blood perfusion in the wound area and promote wound healing [10]. Among them, keratinocyte growth factor (KGF) is a mitogen of a variety of epithelial cells involved in mediating epithelial cell differentiation [11]. It is upregulated after injury and has been shown to play an important role in soft tissue healing [12].

Another member of the FGF family, basic fibroblast growth factor (bFGF), can accelerate angiogenesis and soft tissue regeneration and inhibit scar formation by regulating the degradation and rebuilding of the extracellular matrix, thus effectively promoting wound healing [13].

It has been proved that there is a possibility for iPSCs directly differentiated into endothelial cells [14, 15] or keratinocytes [16, 17]. For inducing the differentiation of MSCs into epidermoid cells, coculture with keratinocytes is a common method [18]. Growth factors such as KGF secreted by keratinocytes can promote the differentiation of MSCs into epidermal cell lines [19, 20]. Barui et al. summarized that cytokines like FGF, EGF, and KGF can be regarded as biochemical cues for MSCs transdifferentiation into epithelial lineage cells [21].

The bFGF-treated MSCs could express higher vascularization markers, such as CD31, thereby facilitating vascularization of the injured area and promoting immune responses [22, 23]. And that when KGF-treated MSCs were applied for repair of burn wounds, skin attachments recovered better than that with untreated MSCs [24].

**Figure 1:**
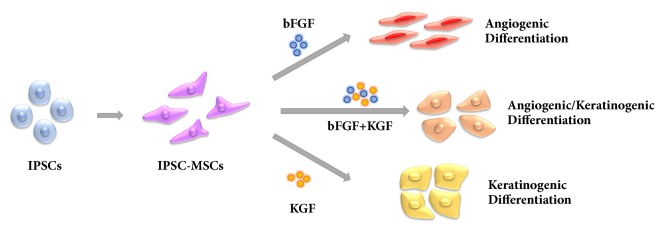
Derivation of iPSC-MSCs from iPSCs and the angiogenic and/or keratinogenic differentiation via bFGF and/or KGF induction.

Plentiful researches about the induction of iPSC-MSCs into some cell lineages, such as osteocytes and cardiomyocytes, have already been reported [3, 25]. However, to the best of our knowledge, there have been no reports about inducing iPSC-MSCs with angiogenic and keratinogenic differentiation potential. In our previous study, iPSC-MSCs have been induced to differentiate into osteoblast-like cells [26]. And in this study, it was expected that iPSC-MSCs could differentiate into epithelial-like and vascular-like cells by the induction of KGF and bFGF. By the induction of bFGF and/or KGF, iPSC-MSCs successfully differentiated into cells with angiogenic and/or keratinogenic differentiation potential (Figure 1). Furthermore, this cell line with angiogenic and keratinogenic differentiation potential provides a promising cell source of for the construction of well vascularized and keratinized tissue engineered skin.

## 2. Materials and Methods

### 2.1. IPSC Culture

The human iPSC ATCC-DYR0100 cell line was purchased from ATCC (ATCC® ACS-1011™, USA). IPSCs were cultured as colonies on a feeder layer of mitotically inactivated murine embryonic fibroblasts (MEF) as previously reported [27]. After proliferation, iPSCs were detached from MEF and dissociated into clumps by incubation with type IV collagenase for 10 min. Dissociated iPSC clumps were collected and resuspended for further use.

### 2.2. Derivation of iPSC-MSCs from iPSCs

IPSC-MSCs were obtained from iPSC according to previous study [28]. In brief, iPSCs were seeded onto gelatin-coated plates at 1×10^4^ cells/cm^2^ in MSC induction media consisting of DMEM-High Glucose (Gibco), 10% defined fetal bovine serum (FBS; Gibco), 1% nonessential amino acids, 1% penicillin-streptomycin, and 5 ng/ml human recombinant bFGF (Protech). Cells were propagated to 80% confluency in a humidified atmosphere at 37°C and 5% CO_2_. For routine expansion, cells were plated at 1×10^4^ cells/cm^2^ and maintained in MSC induction media. Before and after the third and fifth day of iPSCs induction, a light microscopy was used to observe morphological changes occurring during iPSCs differentiation into fibroblast-like cells with 100 times magnification, especially whether they developed a homogeneous fibroblastic morphology

### 2.3. Multipotent Differentiation Potential of iPSC-MSCs

#### 2.3.1. BFGF Induction

IPSC-MSCs were cultured until 70-80% confluence and the original medium was replaced with induction medium. BFGF induction was performed by exposure to 10 ng/ml bFGF, 100 U/ml penicillin, and streptomycin in DMEM medium containing 20% FBS for 2 weeks.

#### 2.3.2. KGF Induction

IPSC-MSCs were cultured until 70-80% confluence and the original medium was replaced with induction medium. KGF induction was performed by exposure to 40 ng/ml KGF, 100 U/ml penicillin, and streptomycin in DMEM medium containing 20% FBS for 2 weeks.

#### 2.3.3. Combined Induction

IPSC-MSCs were cultured until 70-80% confluence and the original medium was replaced with induction medium. Combined induction was performed were grown by exposure to 10 ng/ml KGF and 40 ng/ml bFGF, 100 U/ml penicillin, and streptomycin in DMEM medium containing 20% FBS for 2 weeks.

### 2.4. Flow Cytometry

To confirm the derivation of iPSC-MSCs, surface antigen expression of iPSC-MSCs was characterized via flow cytometry. IPSC-MSCs (passage 5) were harvested by trypsin-ethylenediaminetetraacetic acid (EDTA) and washed with phosphate buffered saline (PBS) containing 0.5% bovine serum albumin (BSA) and then resuspended to ~5×10^5^ cells in 50 *μ*L of PBS containing 0.5% BSA and 2mM EDTA. Cell samples were separately labeled on ice with optimal dilution of fluorescein isothiocyanate-conjugated monoclonal antibodies against CD31, CD44, CD45, and CD90 (Biolegend). After 20min incubation at 4°C, cells were washed with PBS containing 0.5% BSA. Samples were run on a Flow Cytometer (Beckman, MoFlo XDP) instrument. For each analysis, a minimum of 10,000 cells was assayed.

### 2.5. Immunofluorescence Staining

IPSC-MSCs were plated on glass coverslips in 24-well plates at 5×10^5^/well. The following day, cells were serum starved overnight. iPSC-MSCs were fixed in 4% paraformaldehyde, permeabilized with 0.5% Triton X-100, and blocked with 0.5% BSA prior to antibody addition. Indirect immunofluorescence experiments were performed using the KGFR, FGFR, CD31, and K10 monoclonal antibodies (Abcam) overnight at 4°C. Coverslips were then incubated for 60 minutes at room temperature with biotinylated goat anti-rabbit IgG (Abcam) diluted 1:250 in 0.5% BSA. Nuclei were counterstained with 300 nM 4',6-diamidino-2-phenylindole (DAPI, Suobaolai, Beijing). The stained cells were observed with a confocal laser scanning microscope with 100 times magnification (CLSM Nikon, A1),

### 2.6. Statistical Analysis

All results are presented as mean value ± standard deviation. Differences between groups were analyzed by analysis of variance (one-way ANOVA) by statistic software SPSS 22.0.

## 3. Results and Discussion

### 3.1. IPSC-MSC Induction and Identification

**Figure 2:**
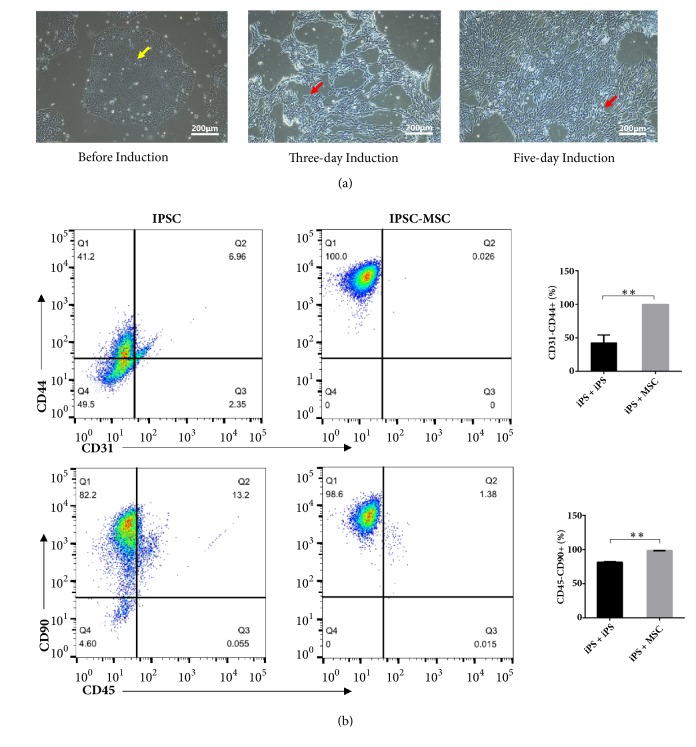
(a) Morphology of iPSCs and iPSC-MSCs observed by a light microscope (×100). The morphology of the iPSC-MSCs resembled elongated spindle shaped cells and differed significantly from the undifferentiated iPSCs. The yellow arrow showed that cell morphology exhibited a round shape, and the red arrows showed that cell morphology changed to a long spindle shape. Scale bar 200*μ*m. (b) Surface antigen expression of CD on undifferentiated iPSCs and iPSC-MSCs by flow cytometry analysis. Flow cytometry analysis showed iPSC-MSCs obviously expressed markers associated with the mesenchymal phenotype (positive for CD44 and CD90; negative for CD31 and CD45) compared to undifferentiated iPSCs. ^*∗∗*^*P*<0.01, one-way ANOVA with Tukey's multiple comparison test.

The shape of the iPSCs was round before induction, and after three-day induction, the cell morphology was changed into a fibroblast-like long spindle type (Figure 2(a)). The flow cytometry results of the iPSC-MSCs (Figure 2(b)) show that the MSC surface markers CD44 and CD90 were expressed to levels greater than 95% in these iPSC-MSCs. On the other hand, the expressions of hematopoietic markers, CD31 and CD45, were negative in the iPSC-MSCs. These results showed that the typical MSC surface markers were consistently and highly expressed in the iPSC-MSCs. Compared with the undifferentiated iPSCs, it could be seen that the iPSCs successfully differentiated into the MSC-like cells with a purity of more than 98% by the induction of MSC medium.

### 3.2. FGFR and KGFR Expression

**Figure 3:**
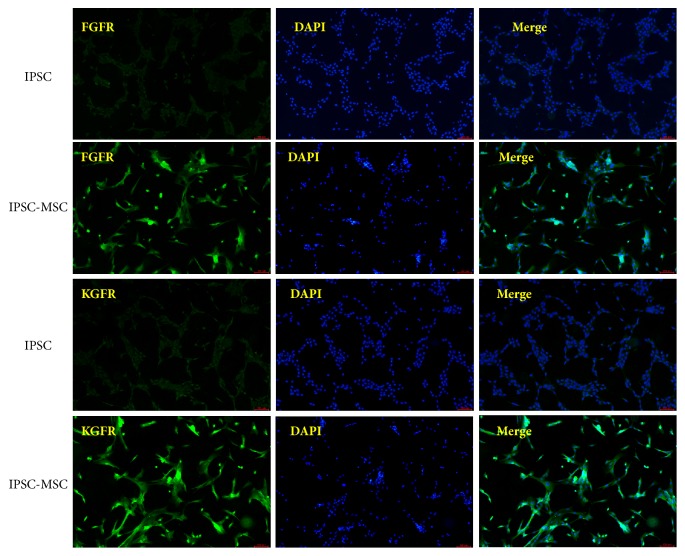
Immunofluorescence staining of KGF receptor and bFGF receptor on iPSCs and iPSC-MSCs (×100). The results showed that the expression of FGFR/KGFR on the cell surface was significantly enhanced after MSC induction.

FGFRs have been shown to be expressed on the surface of MSCs and has an important role for tissue development, such as chondrogenesis and osteogenesis [29, 30]. We detected the expression of KGFR and FGFR on the surface of iPSC-MSCs by immunofluorescent staining.  Figure 3 showed that FGFR and KGFR expression were both positive on the surface of iPSC-MSCs and negative on the surface of iPSCs. Growth factors diffuse out and bind to a specific cell membrane receptors to stimulate a specific signal pathway which further promote MSCs transdifferentiation to a specific cellular lineage [31]. After induction, the expression of FGFR and KGFR in iPSC-MSCs was significantly higher than the undifferentiated iPSCs; thus we speculated that the differentiation of iPSCs into iPSC-MSCs would enhance the response of the cells to bFGF and KGF, while bFGF or KGF induction might not be sufficient to differentiate iPSCs into endothelial cells or keratinocytes.

### 3.3. BFGF or KGF Induction

**Figure 4:**
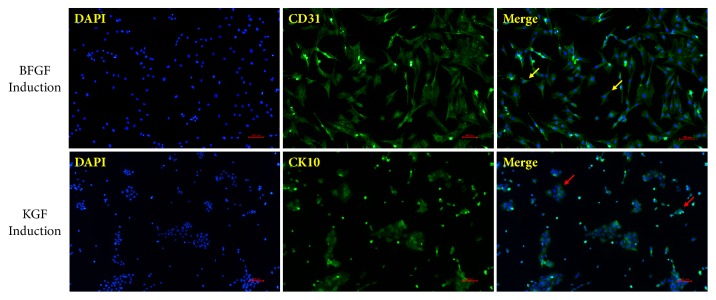
Immunofluorescence staining of DAPI (blue) and CD31/CK10 (green) in iPSC-MSCs after bFGF/KGF induction (×100). After bFGF/KGF induction, CD31/CK10 was significantly expressed in iPSC-MSCs. The yellow arrows indicated that cell morphology still exhibited a MSC-like long spindle type, and the red arrow showed that cell morphology gradually changed from long spindle shape to oblate or irregular shape.

CD31 and CK10 are markers of vascularization and keratinization, respectively [32, 33]. We chose these two markers to detect the vascularization and keratinization of iPSC-MSCs. Immunofluorescence staining showed that CD31 was significantly expressed on the cell surface after bFGF induction, and K10 expression was also markedly in the cytoplasm of cells after KGF induction (Figure 4).

At the same time, we also found that the morphology of iPSC-MSCs induced by KGF changed: the cell morphology gradually changed from long spindle shape to oblate or irregular shape, which was close to the morphology of epithelial cells. Changes in cell morphology indicate iPSC-MSCs differentiation towards epidermal-like cells. However, no obvious morphological change was observed in bFGF-induced iPSC-MSCs, suggesting that bFGF had no significant effect on promoting the differentiation towards epidermal-like cells.

### 3.4. Combined Induction

In order to construct a cell lineage that is more suitable for skin tissue engineering, we further combined the two growth factors together for inducing iPSC-MSCs into a cell lineage with both angiogenic and keratinogenic differentiation potential. Immunofluorescence staining showed that both CD31 and K10 were significantly expressed after bFGF and KGF coinduction.

**Figure 5:**
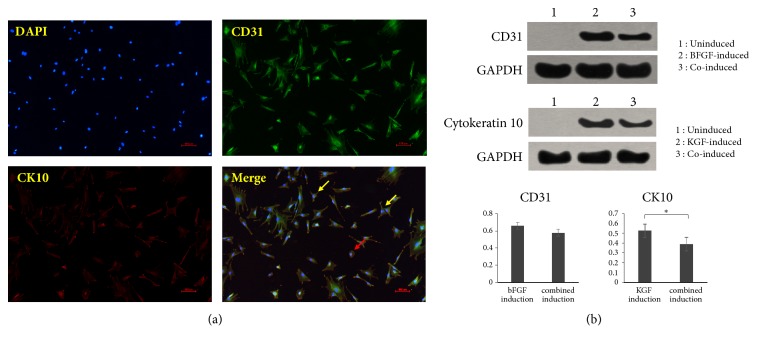
(a) Immunofluorescence staining of DAPI, CD31, and CK10 of iPSC-MSCs after combined induction (×100). (b) Results of Western blot assay, indicating that bFGF or KGF induction could enhance the expression of CD31 or CK10, whereas in the combined induction group, the expression of CD31 and K10 was lower than that of the single induction group. ^*∗*^*P*<0.05. The red arrow indicated that only a small number of cells showed a tendency towards shifting into a round shape after induction; meanwhile the yellow arrow showed that most of the cells still exhibited a MSC-like long spindle type.

However, unlike in Figure 3, only a small number of cells showed a tendency towards shifting into a round shape after induction (Figure 5(a)). Most of the cells still exhibited a MSC-like long spindle type, suggesting that bFGF induction may affect the KGF-induced differentiation of iPSC-MSCs into epidermal-like cells.

Similarly, western blot results (Figure 5(b)) showed that uninduced iPSC-MSCs did not express CD31 or K10 protein, while CD31 and K10 were significantly expressed, respectively, after bFGF or KGF induction. After induction of combination, both CD31 and K10 were significantly expressed, which is consistent with the results of immunofluorescence staining. However, in the combined induction group, the expressions of CD31 and K10 were lower than that of the single induction groups, suggesting that the effects of the two growth factors interfered with each other. In other studies, there were also cases where growth factors interfered with each other when applied in combination. When BMP-2 and bFGF were simultaneously applied to porcine periodontal ligament fibroblasts TesPDL3, the application of BMP-2 inhibited the formation of neovascularization, while the application of bFGF inhibited the expression of osteogenic markers and extracellular mineralization [34]. Cells with strong proliferation ability are usually less differentiated, while with higher differentiated degree, the proliferation activity is always weakened. Therefore, we speculate that the effect of the growth factors on promoting cell proliferation conflicted with the differentiation induced by them, leading to the decreased expression of CD31 and CK10.

## 4. Conclusions

In this study, iPSC-MSCs were successfully differentiated into cells with angiogenic and/or keratinogenic potential via the induction of bFGF and/or KGF. This cell line with angiogenic and keratinogenic differentiation potential provides a promising new source of cells for the construction of well vascularized and keratinized tissue engineered skin. At the same time, we also found that although the induction of bFGF and KGF could increase the expression of vascularization and keratinization markers, respectively, the effects of inducing cell differentiation by the two conflicted with each other, thus more appropriate concentration of growth factors still remained to be explored.

## Data Availability

The pictures used to support the findings of this study are included within the article.
